# Sequence analysis of mitochondrial genome of the false and phantom crane-fly *Ptychoptera qinggouensis* Kang, Yao and Yang, 2013 (Diptera, Ptychopteridae)

**DOI:** 10.1080/23802359.2020.1788452

**Published:** 2020-07-11

**Authors:** Shuo Ma, Zehui Kang

**Affiliations:** Key Lab of Integrated Crop Pest Management of Shandong Province, College of Plant Health and Medicine, Qingdao Agricultural University, Qingdao, China

**Keywords:** Mitochondrial genome, phylogeny, *Ptychoptera*, false and phantom crane-fly

## Abstract

The genus *Ptychoptera* Meigen, 1803 is the largest genus of the family Ptychopteridae with 78 known species. In this study, we report a nearly complete mitochondrial (mt) genome of this genus, which is a circular molecule of more than 15,028 bp. The mt genome contains 13 protein-coding genes, 22 tRNA genes, 2 rRNA genes and a non-coding region. The overall base composition is A (38.1%), T (36.7%), C (14.9%), and G (10.4%), with an AT content of 74.8%. The AT content of N-strand genes (75.7%) is higher than that of the J-strand genes (71.7%). Phylogenetic analysis reveals that the monophyly of Ptychopteridae, Bibiomorpha, Tipulomorpha and Brachycera are strongly supported, and the sister group relationship between Tanyderidae and Ptychopteridae is not supported.

The family Ptychopteridae includes three genera, *Bittacomorpha* Westwood, 1835, *Bittacomorphella* Alexander, 1916, and *Ptychoptera* Meigen, 1803, of which *Ptychoptera* is the largest genus and widely distributed in the world except for the Australasian (Oceanian) region with 78 described species (Kang et al. 2013a, 2013b; Paramonov 2013; Török et al. 2015). More mitochondrial (mt) genomes have been widely used for reconstructing phylogenetic relationships in many insect groups, including the lower Diptera (Beckenbach 2012; Timmermans and Vogler 2012; Wang et al. 2012; Caravas and Friedrich 2013; Li et al. 2013; Li et al. 2015; Wang et al. 2017). There are nearly 700 complete or nearly complete lower dipteran mt genomes available in GenBank, of which only two mt genomes are from Ptychopteridae. Here, we report another nearly complete mt genome of Ptychopteridae.

The specimen of *Ptychoptera qinggouensis* Kang, Yao and Yang, 2013 used in this study was collected from Daqinggou, Kezuohouqi, Neimenggu, China (42°45′47ʺN 122°12′14ʺE, 200 m) and stored in the Entomological Museum of Qingdao Agricultural University, China (No. PTY0001). Qualified DNA samples were pooled for next-generation sequencing library construction following the method proposed by Gillett et al. (2014). The standard PCR reactions were sequenced by primers designed by Simon et al. (2006). BLAST searches were conducted with BioEdit 7.0.5.3 for the bait sequence against mt genome assemblies. The sequence was annotated following the method proposed by Cameron (2014). Maximum-likelihood analysis was conducted by RAxML v7.0.3 (Stamatakis 2006).

The nearly complete mt genome of *P. qinggouensis* (GenBank accession no. MT380468) is 15,028 bp in length. It contains 13 protein-coding genes, 22 tRNA genes, 2 rRNA genes and a non-coding region. The overall base composition is A (38.1%), T (36.7%), C (14.9%), and G (10.4%), with an AT content of 74.8%. It has a weakly positive AT-skew and a negative GC-skew. For protein-coding genes, the AT content of all PCGs is 81.1%. It has a negative AT-skew and a positive GC-skew. The AT content of N-strand genes (75.7%) is higher than that of the J-strand genes (71.7%). The AT content of PCG third codon positions is much higher than that of the first and second codon positions and the AT content of PCG third codon positions of N-strand genes (87.1%) is the highest. PCGs of J-strand genes have negative AT-skew and GC-skew. PCGs of N-strand genes have negative AT-skew and positive GC-skew. For RNA genes, The AT content of tRNA was 76.9% and the AT-skew and GC-skew are positive. The AT content of the 16S rRNA (80.7%) is slightly higher than that of the 12S rRNA (77%). They both have negative AT-skew and a positive GC-skew.

The phylogenetic tree in this study (Figure 1) shows a strong support for the monophyly of the Bibiomorpha (100%), Tipulomorpha (99%) and the Brachycera (100%). The phylogenetic relationship in Tipulomorpha is Trichoceridae + (Pediciidae + (Limoniidae + (Cylindrotomidae + Tipulidae))). The monophyly of the family Ptychopteridae is strongly supported, whereas its phylogenetic position in the lower diptera is undefined.

**Figure 1.**
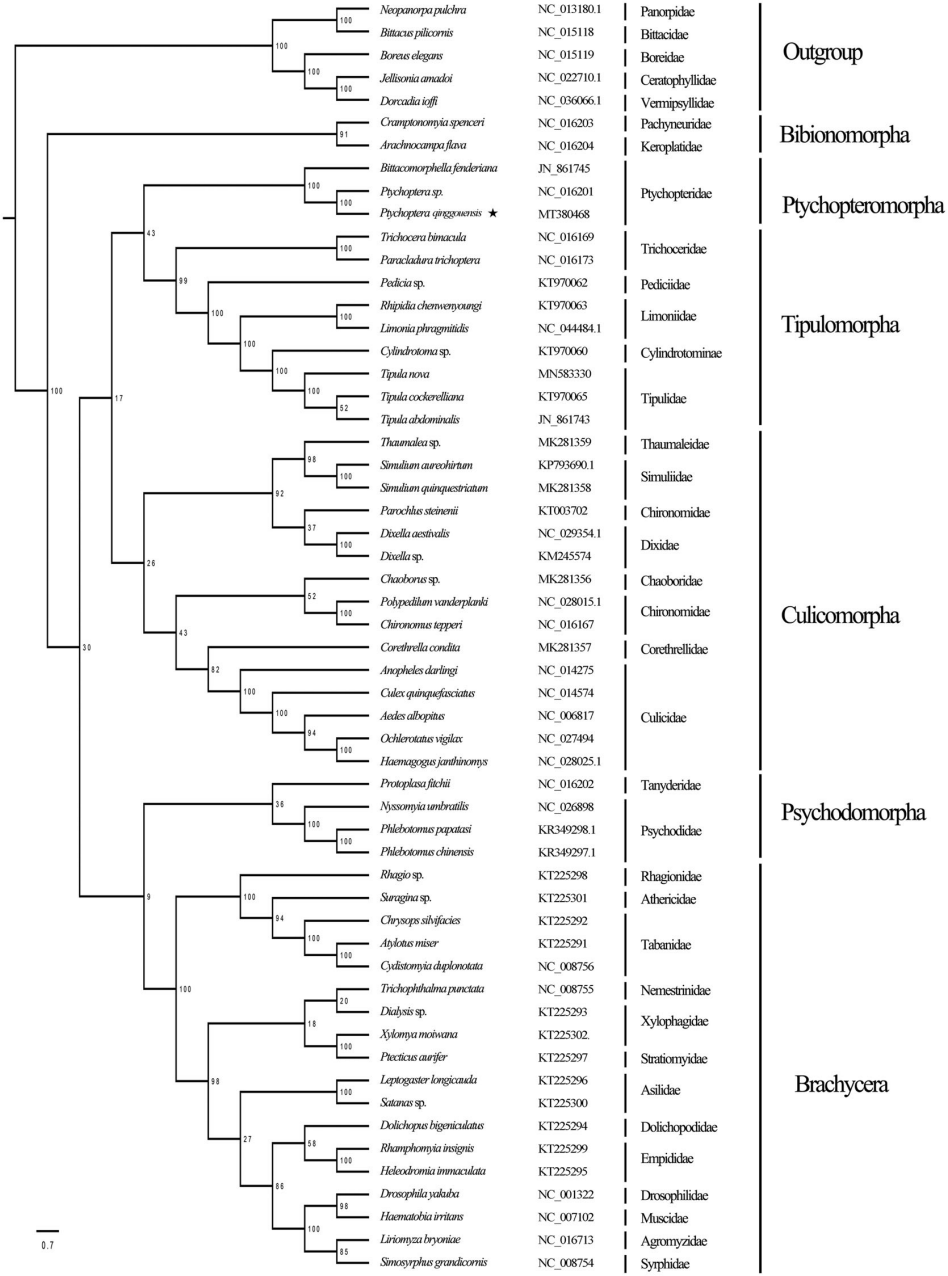
Phylogenetic tree of Nematocera based on whole mitochondrial genomes using maximum-likelihood analysis. Numbers above the branches are bootstrap percentages. GeneBank accession numbers of each species are listed in the tree.

## Data Availability

The data that support the findings of this study are openly available in NCBI at https://www.ncbi.nlm.nih.gov/. GeneBank accession numbers are listed as follows: *Aedes albopitus* (NC_006817), *Anopheles darlingi* (NC_014275), *Arachnocampa flava* (NC_016204), *Atylotus miser* (KT225291), *Bittacomorphella fenderiana* (JN_861745), *Bittacus pilicornis* (NC_015118), *Boreus elegans* (NC_015119), *Chaoborus* sp. (MK281356), *Chironomus tepperi* (NC_016167), *Chrysops silvifacies* (KT225292), *Corethrella condita* (MK281357), *Cramptonomyia spenceri* (NC_016203), *Culex quinquefasciatus* (NC_014574), *Cydistomyia duplonotata* (NC_008756), *Cylindrotoma* sp. (KT970060), *Dialysis* sp. (KT225293), *Dixella aestivalis* (NC_029354.1), *Dixella* sp. (KM245574), *Dolichopus bigeniculatus* (KT225294), *Dorcadia ioffi* (NC_036066.1), *Drosophila yakuba* (NC_001322), *Haemagogus janthinomys* (NC_028025.1), *Haematobia irritans* (NC_007102), *Heleodromia immaculata* (KT225295), *Jellisonia amadoi* (NC_022710.1), *Leptogaster longicauda* (KT225296), *Limonia phragmitidis* (NC_044484.1), *Liriomyza bryoniae* (NC_016713), *Neopanorpa pulchra* (NC_013180.1), *Nyssomyia umbratilis* (NC_026898), *Ochlerotatus vigilax* (NC_027494), *Paracladura trichoptera* (NC_016173), *Parochlus steinenii* (KT003702), *Pedicia* sp. (KT970062), *Phlebotomus chinensis* (KR349297.1), *Phlebotomus papatasi* (KR349298.1), *Polypedilum vanderplanki* (NC_028015.1), *Protoplasa fitchii* (NC_016202), *Ptecticus aurifer* (KT225297), *Ptychoptera qinggouensis* (MT380468), *Ptychoptera* sp. (NC_016201), *Rhagio* sp. (KT225298), *Rhamphomyia insignis* (KT225299), *Rhipidia chenwenyoungi* (KT970063), *Satanas* sp. (KT225300), *Simosyrphus grandicornis* (NC_008754), *Simulium aureohirtum* (KP793690.1), *Simulium quinquestriatum* (MK281358), *Suragina* sp. (KT225301), *Thaumalea* sp. (MK281359), *Tipula abdominalis* (JN_861743), *Tipula cockerelliana* (KT970065), *Tipula nova* (MN583330), *Trichocera bimacula* (NC_016169), *Trichophthalma punctata* (NC_008755) and *Xylomya moiwana* (KT225302).
